# Antibacterial Activity of Essential Oils as Phytotherapeutic Alternatives Against *Aeromonas* spp. from Ornamental Fish

**DOI:** 10.3390/pathogens15080831

**Published:** 2026-08-07

**Authors:** Faik Sertel Seçer

**Affiliations:** 1 Department of Fisheries and Aquaculture, Faculty of Agriculture, Ankara University, 06110 Ankara, Türkiye; sertelsecer@yahoo.com or secer@ankara.edu.tr; 2 Aquaterra Ltd., Ankara University Tecnopolis, 06830 Ankara, Türkiye

**Keywords:** antimicrobial resistance, phytotherapy, *Aeromonas hydrophila*, *Aeromonas veronii*, One Health, aquaculture

## Abstract

*Aeromonas* species are among the most important Gram-negative pathogens in aquaculture and represent a One Health concern, as antibiotic-resistant strains circulate at the interface between ornamental fish, the aquatic environment and humans. This study evaluated the in vitro antibacterial activity of four commercial essential oils from tea tree (*Melaleuca alternifolia*), peppermint (*Mentha piperita*), rosemary (*Rosmarinus officinalis*) and lavandin (*Lavandula hybrida*) against sixteen oxytetracycline-resistant *Aeromonas hydrophila* and *A. veronii* isolated from moribund ornamental fish. All tested bacteria were resistant to oxytetracycline (MIC 8–16 µg/mL). Minimum inhibitory (MIC) and minimum bactericidal (MBC) concentrations were determined by broth microdilution. All EOs inhibited every isolate, but their bactericidal capacity differed markedly. Rosemary oil showed the lowest MIC and MBC ranks and the most consistent values, followed by tea tree and lavandin; all three were bactericidal. Peppermint oil, despite a low MIC, showed a high MBC and was bacteriostatic. These findings indicate that the EOs of rosemary, tea tree and lavandin are promising in vitro candidates for use against oxytetracycline-resistant *Aeromonas* in aquaculture, in line with the One Health approach, and warrant further evaluation.

## 1. Introduction

In aquaculture, *Aeromonas* causes various infections in fish, crustaceans, invertebrates and even humans. More than 35 species belong to the genus *Aeromonas*, and approximately 20 of these species are known to have pathogenic potential [1]. The widespread use of conventional antibiotics in the treatment of infections causes the development of drug-resistant bacterial strains, a decline in the efficacy of drugs, and their accumulation in the environment, thereby endangering both aquatic ecosystems and human health. In fact, it has been reported that in some aquatic environments, a high proportion of bacteria are resistant to at least one antibiotic and that this resistance spreads rapidly between pathogens and the environmental microbiome, posing a persistent threat to aquatic ecosystems [2].

This increase in antibiotic resistance, along with global regulatory pressures within the ‘One Health’ framework, which integrates human, animal and environmental health, has prompted researchers to seek sustainable and biodegradable alternatives, particularly in aquaculture. In this regard, environmentally friendly phytotherapy practices and essential oils (EOs) derived from plants, which have the potential to reduce antibiotic use, are attracting considerable attention as a promising strategy [3,4,5,6,7,8,9,10,11,12,13,14].

EOs are gaining increasing attention due to their natural origin, their multi-target mechanisms that are thought to reduce the likelihood of resistance emerging and their activity against various Gram-positive and Gram-negative fish pathogens isolated from diseased fish [11,12,15,16,17,18]. The antimicrobial effects of EOs are mostly associated with the terpenes, terpenoids and phenolic compounds they contain; it has been reported that these bioactive compounds exert their effect by disrupting the structure of the bacterial cell membrane [7,19,20].

The minimum inhibitory concentration (MIC) and minimum bactericidal concentration (MBC) are important parameters for assessing the antibacterial activity of EOs prior to in vivo experiments. The MIC is the lowest concentration of an antimicrobial agent capable of inhibiting bacterial growth, whereas the MBC is the lowest concentration that kills 99.9% of bacteria. The difference between these two values is clinically relevant in determining treatment strategies and monitoring the development of resistance [21,22].

For this purpose, phytotherapy with EOs derived from medicinal and aromatic plants is gaining prominence, with studies examining their effects on bacterial resistance mechanisms, particularly in contrast to synthetic antimicrobials, taking the lead [23]. Furthermore, it has been reported that the use of EOs, particularly as feed additives to improve fish welfare and health, has led to significant improvements in key parameters such as growth performance, feed conversion ratio and protein utilisation efficiency [16,17,24,25,26].

Although several EOs have been screened against fish pathogens, data specifically on oxytetracycline-resistant *Aeromonas* from ornamental fish remain scarce, and most previous reports rely on the MIC alone without distinguishing bacteriostatic from bactericidal activity. The aim of the present study was to evaluate the in vitro antibacterial activity of four commercial EOs, tea tree (*Melaleuca alternifolia*), peppermint (*Mentha piperita*), rosemary (*Rosmarinus officinalis*) and lavandin (*Lavandula hybrida*), against oxytetracycline-resistant *Aeromonas* from ornamental fish by determining their MIC, MBC and MBC/MIC ratios, thereby distinguishing bactericidal from bacteriostatic activity and assessing their potential as sustainable phytotherapeutic solutions in aquaculture.

## 2. Materials and Methods

### 2.1. Essential Oils

In this study, four EOs tea tree EO (extracted from *M. alternifolia*), peppermint EO (extracted from *M. piperita* L.), rosemary EO (extracted from *R. officinalis* L.), and lavandin EO (extracted from *L. hybrida* L.) were obtained from Botalife, Türkiye.

### 2.2. Bacterial Strains

*Aeromonas* strains were previously isolated from koi (*Cyprinus rubrofuscus*), goldfish (*Carassius auratus*), discus (*Symphysodon aequifasciatus*) and angelfish (*Pterophyllum scalare*), with or without clinical signs and kept in culture collection. Bacteria were identified using MALDI-TOF (Biotyper 3.0; Microflex LT; Bruker Daltonics GmbH, Bremen, Germany; Maldi Biotyper Real-Time Classification (RTC) version.12-13.532 MSP) and VITEK 2 (bioMérieux, Marcy l’Etoile, France) rapid tests and also with biochemical tests according to Buller [1]. The bacterial pathogens and their origins used in the study are given in Table 1.

### 2.3. Antibacterial Assay

The microdilution method was used to assess antibacterial activity according to the Clinical and Laboratory Standards Institute (CLSI) [27]. Bacterial strains were spread on Mueller–Hinton Agar (MHA) (Merck, Cat No. 1.05437.0500) plates. After overnight incubation, bacterial cultures (~1.1 × 10^8^) were transferred to Mueller–Hinton Broth (MHB) (Condalab, Cat.1214.00) and adjusted to McFarland 0.5 with an optical density (OD_600_) of absorbance ~0.1. This suspension was then diluted in MHB so that, after addition to the wells, the final inoculum was approximately ~5 × 10^5^ CFU/mL [28].

Antimicrobial susceptibility to oxytetracycline was determined by broth microdilution and interpreted using the interpretive criteria of the CLSI, with isolates classified as susceptible (≤1 µg/mL), intermediate (2–4 µg/mL) or resistant (≥8 µg/mL) on the basis of the CLSI clinical breakpoints established for *A. salmonicida* [27]. Because EOs are lipophilic, they were emulsified with 1% Tween 20 to obtain a homogeneous mixture with the water-based bacterial suspension, and were tested at final in-well concentrations ranging from 500 to 0.48 mg/mL; oxytetracycline was tested from 256 to 0.125 µg/mL. For each assay a negative control (200 µL MHB, to verify the absence of contamination), a growth control (100 µL culture medium + 100 µL bacterial suspension) and a solvent control (1% Tween 20 without EO, to confirm that the emulsifier alone did not inhibit growth) were included, and all tests were performed in triplicate.

The MIC values were determined in 96-well plates: 100 µL of MHB was dispensed into all wells, 100 µL of the highest EO concentration was added to the first well and serially two-fold diluted along the row, and 100 µL was discarded from the last well so that each well retained 100 µL; finally, 100 µL of bacterial inoculum (~5 × 10^5^ CFU/mL) was added to every well, giving a final volume of 200 µL and a further two-fold dilution of each concentration. Plates were incubated at 22–24 °C for 24 h, and the MIC was read visually as the lowest concentration showing no bacterial growth (turbidity) [29]. To determine the MBC, a 5 µL sample from each well showing no visible growth (i.e., at the MIC and all higher concentrations) was subcultured onto MHA plates with a 48-pin Multi-Blot Replicator and incubated at 22–24 °C for 24–48 h; the MBC was defined as the lowest concentration that killed ≥99.9% of the initial inoculum, yielding no visible colonies on the subculture plate [29,30,31]. All MIC and MBC determinations were carried out in independent triplicates for each EO–isolate combination. The MBC/MIC ratio was then used to distinguish bacteriostatic from bactericidal activity, with a ratio ≤ 4 taken as bactericidal and a ratio > 4 as bacteriostatic [22].

### 2.4. Data Analyses

Due to the ordinal nature of the data, non-parametric methods were employed. Specifically, the Friedman test was used for both MIC and MBC. The magnitude of the oil effect was expressed as Kendall’s coefficient of concordance (W). Dunn’s test based on mean ranks with a Bonferroni adjustment was used for multiple comparisons. Statistical significance was set at *p* < 0.05. All analyses were performed in IBM SPSS Statistics (version 30; IBM Corp., Armonk, NY, USA).

## 3. Results

### 3.1. Findings of Antibacterial Analysis of the EOs

**Figure A1:**
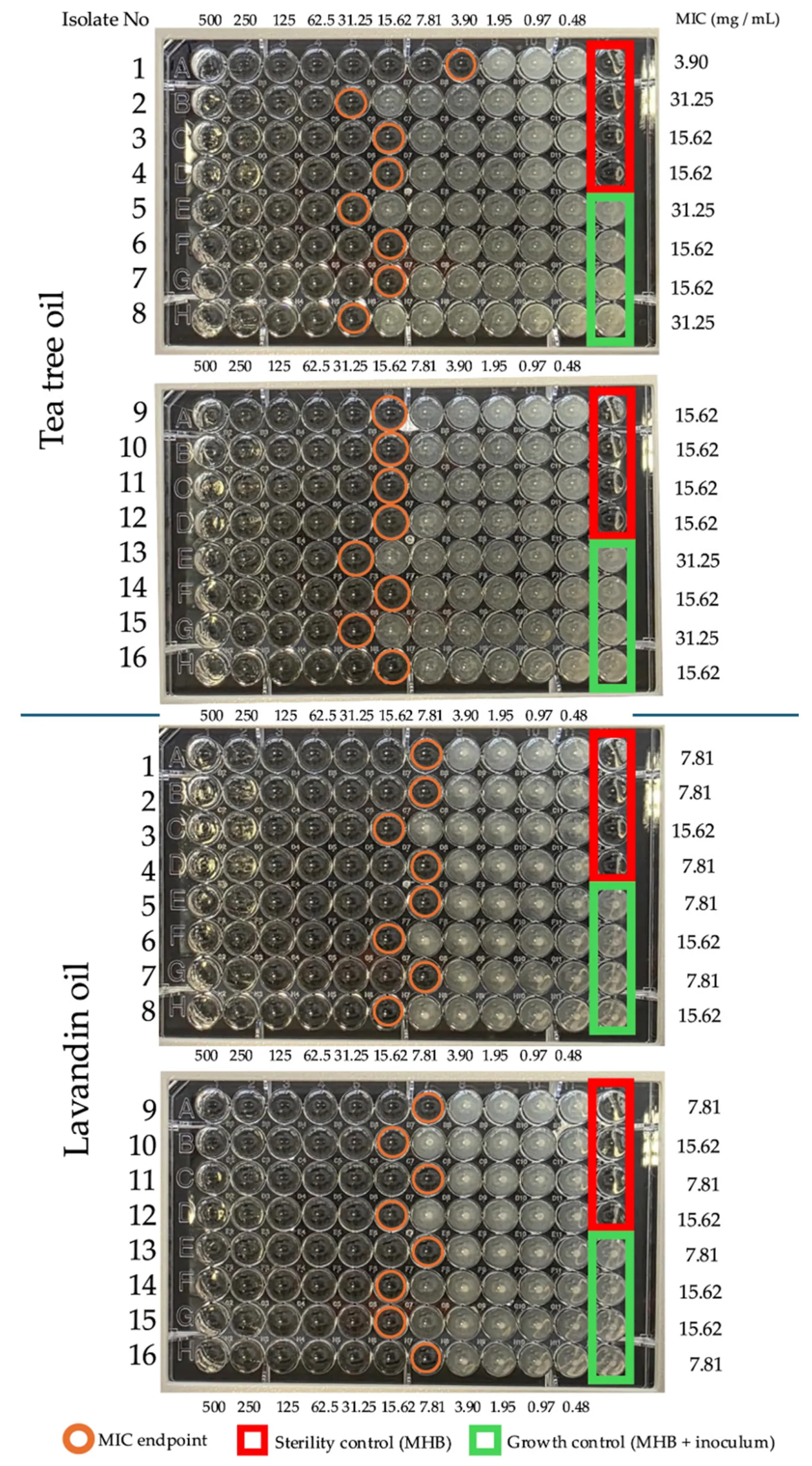
MIC assays results demonstrating the antibacterial activity of tea tree and lavandin essential oils against 16 tested Aeromonas isolates.

In this study, the oxytetracycline MIC values of the isolates ranged from 8 to 16 µg/mL (Table 2); consequently, all field isolates were classified as resistant (R) to the antibiotic. The four EOs were tested against these sixteen *Aeromonas* isolates (No. 1–8, *A. hydrophila*; No. 9–16, *A. veronii*) by broth microdilution, and the MIC, MBC and MBC/MIC ratios with the corresponding bacteriostatic/bactericidal classification are presented in Table 2 and Table A1; representative microdilution plate images for tea tree and lavandin oils were shown in Figure A1.

For all four oils the most frequently observed as modal MIC was 7.81 mg/mL; this value therefore represents the predominant MIC across isolates rather than a fixed value for every isolate, and the full isolate-level ranges are given in Table 2 and Table A1. The oils separated clearly on the basis of their bactericidal activity rather than their MIC. Tea tree and rosemary oils were the most potent, with modal MBCs of 15.62 mg/mL and MBC/MIC ratios of ≤4, and were bactericidal against every isolate; lavandin oil showed an intermediate, stable profile (modal MBC 31.25 mg/mL, MBC/MIC 2–4) and was also bactericidal throughout. In contrast, peppermint oil, despite a comparable modal MIC, required a much higher modal MBC (125 mg/mL; MBC/MIC 8–16) and was bacteriostatic against all isolates.

Rankings across isolates were highly concordant for MBC (Kendall’s W = 0.74) but only moderately so for MIC (W = 0.31) (Table 3). For MBC, rosemary showed the lowest mean rank, followed by tea tree and lavandin, although rosemary and tea tree did not differ significantly (*p* = 0.331); all three were classified as bactericidal (MBC/MIC ≤ 4) in every isolate. Peppermint, despite an MIC comparable to that of lavandin, showed the highest MBC and was bacteriostatic (MBC/MIC 8–16) in all isolates.

### 3.2. Chemical Composition of EOs

The main constituents of the examined EOs were provided by the manufacturer by gas chromatography mass spectrometry (GC/MS), and their major chemical compositions are presented in Table 4.

The main component of tea tree (*M. alternifolia*) oil is terpinen-4-ol, which constitutes 22.37% of the oil. This is followed by γ-terpinene at 16.61%, 2-carene at 11.92%, p-cymene at 8.36%, α-pinene at 6.03%, 1,8-cineole (eucalyptol) at 5.89%, and terpinolene at 5.79%. In peppermint (*M. piperita*) oil, menthol is the most abundant component, making up 29.07%, followed by menthone at 25.61%; menthone and limonene are additionally present at 14.76% and 5.43%, respectively, which is characteristic of the oil’s menthol/menthone-rich profile. In rosemary (*R. officinalis*) oil, 1,8-cineole (eucalyptol) is the major component at 30.06%, followed by α-pinene at 11.51%, camphor at 10.69%, β-pinene at 7.99%, camphene at 7.03%, limonene at 6.45%, and o-cymene at 5.03%. Lavandin (*L. hybrida*) oil is composed of linalool at 26.19%, linalyl acetate at 17.47%, camphor at 9.16%, and 1,8-cineole at 9.13%.

## 4. Discussion

In this study, four commercial EOs were evaluated in vitro against sixteen oxytetracycline-resistant *Aeromonas* isolates (eight *A. hydrophila* and eight *A. veronii*) recovered from moribund ornamental fish. Although all four oils suppressed growth at broadly similar concentrations, with the MIC remaining close to 7.81 mg/mL irrespective of the oil and only moderate agreement in the isolate-level MIC ranking (Kendall’s W = 0.31), their bactericidal activity differed significantly (Friedman χ^2^(3) = 35.67, *p* < 0.001), and the isolate-level ranking of the oils by MBC was highly consistent (Kendall’s W = 0.74). Rosemary, tea tree and lavandin were bactericidal against every isolate, with an MBC/MIC ratio of 4 or below, whereas peppermint, despite inhibiting growth at the same low concentration, required four- to sixteen-fold higher concentrations to kill and therefore remained bacteriostatic throughout, with an MBC/MIC ratio of 8 to 16. This differentiation between growth inhibition and bactericidal activity suggests that MIC alone may underestimate an agent’s bactericidal potential in vitro, and should be interpreted alongside the MBC.

Bacteriostatic agents merely suppress bacterial replication, whereas bactericidal agents actively kill. Because the MIC reflects only the concentration needed to inhibit growth, it cannot by itself distinguish a bacteriostatic from a bactericidal agent; this distinction requires the MBC. An agent that merely suppresses replication, as peppermint oil did, leaves viable bacteria that can resume growth once the compound is diluted or metabolized. For these reasons, the MBC and the derived MBC/MIC ratio, in addition to the MIC, should be considered when screening EOs against *Aeromonas* [22]; on this basis, rosemary, tea tree and lavandin qualify as promising leads. From a practical perspective, the distinction between bacteriostatic and bactericidal agents were significant. A bacteriostatic agent relies on a functional host immune response to eliminate the surviving bacteria that have ceased growing. However, any dilution of the agent in the open water of a culture system may permit bacterial regrowth. In ornamental aquaculture, where precise dosing is challenging and fish often experience immunocompromise or stress, the bactericidal oils, such as rosemary, tea tree, or lavandin, is preferable to a solely bacteriostatic agent like peppermint.

These differences in bactericidal activity are consistent with the oils’ chemical profiles determined by GC-MS. Rosemary oil was dominated by eucalyptol, a membrane-active antibacterial compound [32,33]. Tea tree oil, which exhibited bactericidal activity, is characterized by its high terpinen-4-ol content, which is primarily responsible for its antibacterial efficacy, together with γ-terpinene and p-cymene, a composition typical of the terpinen-4-ol chemotype [34]. Lavandin oil, dominated by linalool and linalyl acetate, also acted bactericidally, in line with the reported membrane-disrupting activity of linalool. In contrast, peppermint oil was rich in menthol and menthone; although these compounds effectively restricted growth, they were comparatively less lethal, which accords with the bacteriostatic profile and with reports that EOs characterized by a high concentration of menthol are strongly inhibitory but variably bactericidal [35].

The results were consistent with earlier studies of EOs against *Aeromonas*. Kot et al. [36] reported bactericidal activity for several plant-derived agents against *Aeromonas* strains from fish and based their bacteriostatic/bactericidal classification on the MBC/MIC ratio. Hudecová et al. [16] similarly found EOs including tea tree and peppermint to be active against *A. hydrophila* and *A. veronii* from rainbow trout, distinguishing bactericidal from bacteriostatic effects, in agreement with the present profiles. Comparable bactericidal activity of EOs against *Aeromonas* has also been documented by Starliper et al. [37] and Baba [15], while Souza et al. [34] showed that tea tree oil exerts a bactericidal effect against *A. hydrophila* in vivo in silver catfish. The concordance of these findings across independent isolate collections reinforces the view that EOs constitute a viable source of antibacterial activity against this genus [19]. All isolates in this study were resistant to oxytetracycline; this indicates that the use of antibiotics in intensive ornamental fish farming increases the likelihood of resistant *Aeromonas* isolates emerging. Such resistance is widely regarded as part of the environment–animal–human interface within the One Health framework. That all tested isolates were resistant to oxytetracycline is in keeping with the growing reports of antimicrobial resistance among *Aeromonas* from aquaculture [38,39,40] and highlights the requirement for sustainable alternatives within the ‘One Health’ framework [13]. Notably, the bacteriostatic profile of peppermint oil observed in the study contrasts with reports in which *Mentha* EOs were bactericidal against *Aeromonas*, Chagas et al. [35], with MIC and MBC values coinciding at 1.25–16.7 mg/mL, depending on the strain. This discrepancy most likely reflects differences in chemotype and in the menthol/menthone balance of the oil, as well as differences in inoculum density and endpoint criteria between studies. It reinforces that the antibacterial property of an EO is condition and chemotype-dependent rather than an intrinsic, transferable property, and that bacteriostatic versus bactericidal classifications should always be interpreted in this study of the specific oil batch and assay used.

The bactericidal activity of rosemary, tea tree, and lavandin oils against these resistant isolates is notable, but the effective concentrations must be viewed realistically. The EO MIC values were roughly three orders of magnitude higher, on a mass basis, than the oxytetracycline concentrations tested (µg/mL range), a gap that is commonly reported for EOs and that has direct implications for aqueous delivery, cost and organoleptic effects in a culture system. This constraint was partly offset by their natural origin, their multi-target mode of action, which is less prone to selecting for resistance, and their lower environmental persistence [4,7]; nevertheless, it means the oils are more realistically positioned as leads for formulation and combination approaches than as direct high-dose replacements for antibiotics.

The present study has several strengths. Antibacterial activity was assessed not by MIC alone but together with the MBC and the derived MBC/MIC ratio, allowing for bacteriostatic and bactericidal effects to be separated; the isolate-level ranking of the oils by MBC was highly consistent across two *Aeromonas* species; and all isolates were oxytetracycline-resistant field strains, so the oils were tested against a clinically relevant, resistant background rather than reference strains alone.

Several methodological limitations should be considered. First, the data are entirely in vitro; broth microdilution measures activity against bacteria in a nutrient-rich medium and does not capture in vivo pharmacokinetics, host toxicity, host immune responses, biofilm behaviour, or efficacy when the oils are delivered in feed or water, so their therapeutic value in fish remains to be established. Importantly, MIC and MBC values quantify intrinsic antibacterial potency under standardised laboratory conditions and do not, by themselves, demonstrate therapeutic efficacy, safety or bioavailability in live fish; accordingly, the present results should not be interpreted as evidence of clinical effectiveness, but only as a first, in vitro indication of antibacterial potential. Second, because the oils are lipophilic, they were solubilised with 1% Tween 20; this emulsifier can influence oil dispersion and thus the apparent MIC and MBC, so the values reported were conditional on this carrier system. Third, the chemical composition was determined from a single GC-MS analysis of one commercial batch per oil; because EO composition varies substantially with chemotype, batch and supplier, the composition-activity relationships discussed above should be regarded as informative and hypothesis-generating rather than definitive. Establishing a causal link between individual constituents and bactericidal activity would require testing purified compounds and multiple batches per oil. Fourth, the study comprised sixteen isolates of two *Aeromonas* species from ornamental fish of a single collection, and all assays were performed in a single laboratory without inter-laboratory replication, which limits generalization to other species, hosts and regions. Although sixteen isolates of two *Aeromonas* species provide a reasonable preliminary dataset, a larger panel spanning additional *Aeromonas* species, host fish and geographic sources were ideally with multi-laboratory testing and would be required before the observed activity ranking can be considered representative of the genus as a whole. Finally, the four oils differed only marginally in MIC, with central values largely tied at 7.81 mg/mL and only moderate concordance in the between-oil MIC ranking (Kendall’s W = 0.31); the discriminatory findings therefore rest primarily on the MBC and the derived bacteriostatic/bactericidal classification. Taken together, these limitations indicate that the present results should be regarded as a preliminary in vitro screen, and that the tested oils are best viewed as candidates for optimization or combination strategies rather than as stand-alone treatments. Such strategies could include nanoencapsulation approaches such as nanoemulsions or liposomes, together with carrier optimization to counter the lipophilicity and volatility of the oils and to improve their aqueous solubility and stability, or combination with conventional antibiotics to screen for possible synergy. Their potential cytotoxicity toward host cells would likewise need to be established before any practical application. Confirmation through in vivo efficacy and toxicity trials, together with standardization of oil composition, would be required before practical application in aquaculture could be considered.

## 5. Conclusions

This study demonstrates that EOs from tea tree, rosemary and lavandin exhibit bactericidal properties, while peppermint oil demonstrates a bacteriostatic effect against oxytetracycline-resistant *A. hydrophila* and *A. veronii* isolated from ornamental fish. Among the oils tested, rosemary was the most consistent, and together with tea tree and lavandin it acted bactericidally, whereas peppermint only inhibited growth. Overall, these in vitro findings identify rosemary, tea tree and lavandin as promising early-stage in vitro phytotherapeutic candidates that merit further investigation against oxytetracycline resistant *Aeromonas*; they do not yet establish clinical usefulness, and controlled in vivo trials of efficacy and safety are needed before any application can be recommended, in line with the One Health approach. Beyond its applied value, such an in vitro screen aligns with the principles of the 3Rs (Replacement, Reduction, Refinement) and with European legislation on the protection of animals used for scientific purposes [41], which explicitly encourages the use of validated non-animal methods wherever they can answer the scientific question. By narrowing a panel of candidate oils before any in vivo challenge, this prescreening strategy reduces the number of fish required in subsequent trials and supports a more ethical and resource-efficient path toward phytotherapeutic development in aquaculture.

## Figures and Tables

**Table 1:** Bacterial pathogens and their origins.

No	Strain	Bacteria	Origin	Source
1	KSAh24-09	*A. hydrophila*	Koi—*Cyprinus rubrofuscus*	Skin Ulcer
2	KSAh25-11			Skin Ulcer
3	KKAh25-16			Kidney
4	JKAh24-04		Goldfish—*Carassius auratus*	Kidney
5	JKAh24-09			Kidney
6	JKAh25-18			Kidney
7	D-Ah24-02		Discus—*Symphysodon aequifasciatus*	Dead fish
8	DSAh25-05			Skin Ulcer
9	KKAv24-13	*A. veronii*	Koi—*Cyprinus rubrofuscus*	Kidney
10	KKAv25-16			Kidney
11	KKAv25-18			Kidney
12	M-Av25-18		Angelfish—*Pterophyllum scalare*	Dead fish
13	MSAv25-22			Skin Ulcer
14	D-Av25-15		Discus—*Symphysodon aequifasciatus*	Dead fish
15	D-Av25-14			Dead fish
16	D-Av25-21			Dead fish

**Table 2:** MIC and MBC values, MBC/MIC ratios and bacteriostatic/bactericidal classification of EOs (mg/mL).

Isolate		Tea Tree Oil				Peppermint Oil				Rosemary Oil				Lavandin Oil				OTC
Species	No	MIC	MBC	MBC/MIC	BC/BS	MIC	MBC	MBC/MIC	BC/BS	MIC	MBC	MBC/MIC	BC/BS	MIC	MBC	MBC/MIC	BC/BS	MIC
*A. hydrophila*	1	3.90	15.62	4	BC	7.81	62.50	8	BS	7.81	15.62	2	BC	7.81	31.25	4	BC	8
	2	31.25	125	4	BC	7.81	62.50	8	BS	3.90	15.62	4	BC	7.81	31.25	4	BC	16
	3	15.62	31.25	2	BC	7.81	125	16	BS	3.90	15.62	4	BC	15.62	31.25	2	BC	16
	4	7.81	15.62	2	BC	7.81	62.50	8	BS	7.81	15.62	2	BC	7.81	31.25	4	BC	8
	5	15.62	62.50	4	BC	7.81	125	16	BS	7.81	15.62	2	BC	7.81	31.25	4	BC	16
	6	7.81	15.62	2	BC	15.62	125	8	BS	7.81	31.25	4	BC	15.62	31.25	2	BC	16
	7	7.81	31.25	4	BC	7.81	125	16	BS	3.90	15.62	4	BC	7.81	31.25	4	BC	16
	8	15.62	15.62	1	BC	7.81	125	16	BS	7.81	15.62	2	BC	15.62	31.25	2	BC	16
*A. veronii*	9	15.62	15.62	1	BC	7.81	125	16	BS	7.81	15.62	2	BC	7.81	31.25	4	BC	8
	10	7.81	15.62	2	BC	7.81	125	16	BS	7.81	15.62	2	BC	15.62	31.25	2	BC	16
	11	7.81	15.62	2	BC	7.81	125	16	BS	7.81	15.62	2	BC	7.81	31.25	4	BC	16
	12	7.81	31.25	4	BC	7.81	125	16	BS	7.81	15.62	2	BC	15.62	31.25	2	BC	16
	13	31.25	125	4	BC	7.81	62.50	8	BS	3.90	15.62	4	BC	7.81	31.25	4	BC	8
	14	15.62	31.25	2	BC	7.81	62.50	8	BS	7.81	15.62	2	BC	15.62	31.25	2	BC	16
	15	15.62	15.62	1	BC	7.81	62.50	8	BS	7.81	15.62	2	BC	15.62	31.25	2	BC	16
	16	7.81	31.25	4	BC	15.62	125	8	BS	7.81	15.62	2	BC	7.81	31.25	4	BC	8

OTC: oxytetracycline (µg/mL); BC: bactericidal; BS: bacteriostatic. MIC and MBC values are the modal values (mg/mL) of three independent replicates.

**Table 3:** Pairwise comparisons among EOs (Bonferroni-adjusted *p*-values) for MIC and MBC.

Pairwise Comparison	MIC, Adj. *p*	MBC, Adj. *p*
Tea tree vs. Peppermint	1.000	0.001
Tea tree vs. Rosemary	0.068	0.331
Tea tree vs. Lavandin	1.000	1.000
Peppermint vs. Rosemary	1.000	<0.001
Peppermint vs. Lavandin	1.000	0.037
Rosemary vs. Lavandin	0.045	0.024

Adjusted *p*-values from Dunn’s pairwise procedure based on Friedman mean ranks with Bonferroni correction. *p* < 0.05 indicates a significant difference. Friedman test for MIC: χ^2^(3) = 14.64, *p* = 0.002, Kendall’s W = 0.31; for MBC: χ^2^(3) = 35.67, *p* < 0.001, Kendall’s W = 0.74.

**Table 4:** Major chemical composition of the EOs (>5%).

Essential Oil	Compound	CAS Number	Conc. (%)
Tea tree (*M. alternifolia*)	Terpinolene	586-62-9	5.79
	Eucalyptol (1,8-cineole)	470-82-6	5.89
	α-Pinene	7785-70-8	6.03
	p-Cymene	99-87-6	8.36
	2-Carene	554-61-0	11.92
	γ-Terpinene	99-85-4	16.61
	Terpinen-4-ol	562-74-3	22.37
Peppermint (*M. piperita*)	Limonene	5989-27-5	5.43
	Menthone	89-80-5	14.76
	L-Menthone	14073-97-3	25.61
	Menthol	89-78-1	29.07
Rosemary (*R. officinalis*)	o-Cymene	527-84-4	5.03
	Limonene	5989-27-5	6.45
	Camphene	5794-04-7	7.03
	β-Pinene	127-91-3	7.99
	Camphor	464-49-3	10.69
	α-Pinene	7785-26-4	11.51
	Eucalyptol (1,8-cineole)	470-82-6	30.06
Lavandin (*L. hybrida*)	Eucalyptol (1,8-cineole)	470-82-6	9.13
	Camphor	464-49-3	9.16
	Linalyl acetate	115-95-7	17.47
	Linalool	78-70-6	26.19

## Data Availability

The data presented in this study are available on request from the corresponding author.

## Abbreviations

The following abbreviations are used in this manuscript:

MIC	Minimum inhibitory concentration
MBC	Minimum bactericidal concentration
EO	Essential oil
CLSI	Clinical and Laboratory Standards Institute
MHA	Mueller–Hinton Agar
MHB	Mueller–Hinton Broth
OTC	Oxytetracycline
BC	Bactericidal
BS	Bacteriostatic
GC/MS	Gas chromatography/mass spectrometry

## Appendix A

**Table A1:** Individual MIC and MBC values (mg/mL) of four EOs against 16 oxytetracycline-resistant *Aeromonas* isolates. Values of three independent replicates, the modal value, the MBC/MIC ratio and the bacteriostatic/bactericidal classification are shown for each isolate.

Oil	Iso.	MIC Replicates	MIC Mode	MBC Replicates	MBC Mode	MBC/MIC	Class.
**Tea tree**	1	3.90, 3.90, 1.95	**3.90**	15.62, 15.62, 7.81	**15.62**	4	**BC**
	2	31.25, 31.25, 15.62	**31.25**	125, 125, 62.50	**125**	4	**BC**
	3	7.81, 15.62, 15.62	**15.62**	31.25, 31.25, 62.50	**31.25**	2	**BC**
	4	15.62, 7.81, 7.81	**7.81**	31.25, 15.62, 15.62	**15.62**	2	**BC**
	5	15.62, 31.25, 15.62	**15.62**	62.50, 125, 62.50	**62.50**	4	**BC**
	6	7.81, 15.62, 7.81	**7.81**	15.62, 62.50, 15.62	**15.62**	2	**BC**
	7	15.62, 7.81, 7.81	**7.81**	31.25, 31.25, 62.50	**31.25**	4	**BC**
	8	15.62, 31.25, 15.62	**15.62**	31.25, 15.62, 15.62	**15.62**	1	**BC**
	9	7.81, 15.62, 15.62	**15.62**	31.25, 15.62, 15.62	**15.62**	1	**BC**
	10	15.62, 7.81, 7.81	**7.81**	15.62, 31.25, 15.62	**15.62**	2	**BC**
	11	7.81, 15.62, 7.81	**7.81**	31.25, 15.62, 15.62	**15.62**	2	**BC**
	12	15.62, 7.81, 7.81	**7.81**	62.50, 31.25, 31.25	**31.25**	4	**BC**
	13	31.25, 31.25, 15.62	**31.25**	125, 125, 62.50	**125**	4	**BC**
	14	7.81, 15.62, 15.62	**15.62**	31.25, 31.25, 62.50	**31.25**	2	**BC**
	15	15.62, 31.25, 15.62	**15.62**	15.62, 62.50, 15.62	**15.62**	1	**BC**
	16	7.81, 15.62, 7.81	**7.81**	31.25, 31.25, 31.25	**31.25**	4	**BC**
**Peppermint**	1	15.62, 7.81, 7.81	**7.81**	125, 62.50, 62.50	**62.50**	8	**BS**
	2	7.81, 15.62, 7.81	**7.81**	62.50, 125, 62.50	**62.50**	8	**BS**
	3	15.62, 7.81, 7.81	**7.81**	125, 125, 125	**125**	16	**BS**
	4	7.81, 15.62, 7.81	**7.81**	62.50, 125, 62.50	**62.50**	8	**BS**
	5	7.81, 15.62, 7.81	**7.81**	125, 125, 125	**125**	16	**BS**
	6	7.81, 15.62, 15.62	**15.62**	125, 125, 125	**125**	8	**BS**
	7	7.81, 15.62, 7.81	**7.81**	62.50, 125, 125	**125**	16	**BS**
	8	15.62, 7.81, 7.81	**7.81**	125, 125, 62.50	**125**	16	**BS**
	9	7.81, 15.62, 7.81	**7.81**	125, 125, 125	**125**	16	**BS**
	10	7.81, 15.62, 7.81	**7.81**	125, 125, 125	**125**	16	**BS**
	11	7.81, 15.62, 7.81	**7.81**	125, 125, 125	**125**	16	**BS**
	12	7.81, 7.81, 7.81	**7.81**	62.50, 125, 125	**125**	16	**BS**
	13	7.81, 7.81, 7.81	**7.81**	125, 62.50, 62.50	**62.50**	8	**BS**
	14	15.62, 7.81, 7.81	**7.81**	62.50, 125, 62.50	**62.50**	8	**BS**
	15	7.81, 15.62, 7.81	**7.81**	62.50, 125, 62.50	**62.50**	8	**BS**
	16	7.81, 15.62, 15.62	**15.62**	125, 125, 125	**125**	8	**BS**
**Rosemary**	1	7.81, 3.90, 7.81	**7.81**	15.62, 15.62, 31.25	**15.62**	2	**BC**
	2	3.90, 3.90, 3.90	**3.90**	15.62, 15.62, 15.62	**15.62**	4	**BC**
	3	7.81, 3.90, 3.90	**3.90**	31.25, 15.62, 15.62	**15.62**	4	**BC**
	4	7.81, 3.90, 7.81	**7.81**	15.62, 15.62, 31.25	**15.62**	2	**BC**
	5	7.81, 3.90, 7.81	**7.81**	15.62, 15.62, 15.62	**15.62**	2	**BC**
	6	7.81, 7.81, 3.90	**7.81**	31.25, 31.25, 15.62	**31.25**	4	**BC**
	7	7.81, 3.90, 3.90	**3.90**	15.62, 15.62, 15.62	**15.62**	4	**BC**
	8	7.81, 3.90, 7.81	**7.81**	31.25, 15.62, 15.62	**15.62**	2	**BC**
	9	7.81, 7.81, 3.90	**7.81**	15.62, 15.62, 15.62	**15.62**	2	**BC**
	10	7.81, 3.90, 7.81	**7.81**	31.25, 15.62, 15.62	**15.62**	2	**BC**
	11	7.81, 3.90, 7.81	**7.81**	31.25, 15.62, 15.62	**15.62**	2	**BC**
	12	7.81, 7.81, 3.90	**7.81**	15.62, 15.62, 15.62	**15.62**	2	**BC**
	13	7.81, 3.90, 3.90	**3.90**	31.25, 15.62, 15.62	**15.62**	4	**BC**
	14	7.81, 3.90, 7.81	**7.81**	15.62, 15.62, 15.62	**15.62**	2	**BC**
	15	7.81, 3.90, 7.81	**7.81**	15.62, 15.62, 15.62	**15.62**	2	**BC**
	16	7.81, 7.81, 3.90	**7.81**	31.25, 15.62, 15.62	**15.62**	2	**BC**
**Lavandin**	1	7.81, 15.62, 7.81	**7.81**	31.25, 62.50, 31.25	**31.25**	4	**BC**
	2	7.81, 7.81, 7.81	**7.81**	31.25, 31.25, 31.25	**31.25**	4	**BC**
	3	15.62, 15.62, 7.81	**15.62**	31.25, 62.50, 31.25	**31.25**	2	**BC**
	4	7.81, 15.62, 7.81	**7.81**	31.25, 31.25, 31.25	**31.25**	4	**BC**
	5	15.62, 7.81, 7.81	**7.81**	62.50, 31.25, 31.25	**31.25**	4	**BC**
	6	15.62, 7.81, 15.62	**15.62**	31.25, 31.25, 62.50	**31.25**	2	**BC**
	7	15.62, 7.81, 7.81	**7.81**	31.25, 31.25, 31.25	**31.25**	4	**BC**
	8	15.62, 7.81, 15.62	**15.62**	31.25, 31.25, 31.25	**31.25**	2	**BC**
	9	7.81, 7.81, 7.81	**7.81**	62.50, 31.25, 31.25	**31.25**	4	**BC**
	10	15.62, 15.62, 7.81	**15.62**	31.25, 31.25, 31.25	**31.25**	2	**BC**
	11	15.62, 7.81, 7.81	**7.81**	31.25, 31.25, 31.25	**31.25**	4	**BC**
	12	15.62, 7.81, 15.62	**15.62**	31.25, 31.25, 31.25	**31.25**	2	**BC**
	13	7.81, 7.81, 7.81	**7.81**	31.25, 31.25, 31.25	**31.25**	4	**BC**
	14	15.62, 15.62, 7.81	**15.62**	62.50, 31.25, 31.25	**31.25**	2	**BC**
	15	15.62, 7.81, 15.62	**15.62**	31.25, 31.25, 31.25	**31.25**	2	**BC**
	16	7.81, 7.81, 7.81	**7.81**	31.25, 31.25, 31.25	**31.25**	4	**BC**

MIC, minimum inhibitory concentration; MBC, minimum bactericidal concentration; modal, value recorded in ≥2 of 3 replicates (median where all three differed); BC, bactericidal (MBC/MIC ≤ 4); BS, bacteriostatic (MBC/MIC > 4). Iso., isolate number.
